# Be the change you seek in science

**DOI:** 10.1186/s12915-019-0647-3

**Published:** 2019-03-27

**Authors:** Michael P. Milham, Arno Klein

**Affiliations:** 1 Center for the Developing Brain, Child Mind Institute, New York, NY USA; 2 Center for Biomedical Imaging and Neuromodulation, Nathan S. Kline Institute for Psychiatric Research, New York, NY USA; 3 MATTER Lab, Child Mind Institute, New York, NY USA

## Abstract

Few would argue that science is better done in silos, with no transparency or sharing of methods and resources. Yet scientists and scientific stakeholders (e.g., academic institutions, funding agencies, journals) alike continue to find themselves at a relative impasse in the implementation of open science practices, slowing advancement and inadvertently perpetuating ongoing crises surrounding reproducibility. The present commentary draws attention to critical gaps in the current scientific ecosystem that perpetuate closed science practices and divide the community on how to best move forward. It also challenges scientists as individuals to improve the quality of their science by incorporating open practices in their everyday work, and provides a starter list of steps that any researcher can take to be the change they seek.

The drive towards “open science”—making data and tools free and accessible to researchers in an effort to accelerate discovery—remains deeply controversial despite more than a decade of discussions and debates. Commentaries and calls for action reinforce talking points for both sides of the debate [1, 2]. Open science advocates cite crises in reproducibility, transparency, and rigor that they say can only be addressed through open sharing of materials, methods, data, results, and software. They also highlight the growing needs of “big data” research methods, which in many cases require the sharing and aggregation of independently collected datasets to achieve the sample sizes needed in a timely manner. Open science skeptics express concerns about the potential loss of competitive advantage for their labs and trainees, as well as the various logistical demands of sharing software, data, results, and knowledge (such as documentation, organization, curation, privacy protection, user support). They also note that sharing efforts commonly go uncited and unrewarded by institutions and funding agencies, and they continue to raise questions about the value of shared data. Given the various promises and pitfalls associated with open science, there is an understandable resistance to change, creating what appears to be an impasse.

The debate regarding open science is particularly prominent in the brain imaging community for reasons that, at a high level, may mirror polarizing factors across all of science. For example, one possible explanation is that the exorbitant costs of brain imaging research and the stakes raised by large grant opportunities divide researchers into camps of haves and have-nots. Those with access to data and computational resources want to maximize their return on investment and may be less willing to share. Those without such access may wish to share their expertise to benefit from others’ investments. In this scenario, data and computational resources are capital; unequal access to the amount and quality of data one can acquire, or the data analyses one can perform, dictate who can do scientific research. In some cases, underpowered studies relying on small sample sizes have unwittingly contributed to what has become a much-publicized reproducibility crisis in science [3]. If efforts are not made to encourage or enforce greater sharing, the divide between the haves and have-nots may only grow worse, as data acquisition and analysis costs continue to rise.

It is not simply unequal access to funds and facilities that provokes this divide, but a fragmented community. Like many fields, brain imaging is highly interdisciplinary, requiring expertise spanning engineering, physics, computer science, statistics, psychology, physiology, neuroscience, medicine, and so on. The individual who designs and leads a brain imaging study (e.g., psychologist, psychiatrist) is probably not the most qualified to analyze the data, and vice versa. As highlighted in recent articles [4], the increasing scale and sophistication of the questions of modern neuroscience require data generators, tool makers, and data users to all work closely together. Given this reality, large research institutions have a distinct advantage over smaller organizations in that they can assemble highly interdisciplinary teams. For such institutions with self-contained systems of reward and promotion, there may be little motivation to seek outside help. Unless a culture emerges or steps are taken to cultivate greater cross-institutional collaboration, this fragmentation will continue to define who does scientific research and who does not.

What will it take to bridge this divide? In order to begin addressing this question, we must acknowledge that individuals who would support and benefit from open science often struggle in isolation against great institutional and societal challenges. Acknowledging the magnitude of the challenges at hand, many have turned to key organizing bodies in the community, such as publishers, academic institutions, funding agencies, and professional associations to provide guidance, or to set and enforce standards on their behalf. However, turning to organizing bodies for change presupposes that they not only have everyone’s interests in mind, but that they have the organizational ability and authority to either guide and influence or enact and enforce a resolution. This is not always the case. As noted in a recent editorial [5], when mandates such as post-publication availability of data are put in place, they often go ignored and unenforced. For many, incentives represent an appealing alternative to mandates. In this regard, funding agencies have created a number of funding opportunities to facilitate the sharing of previously collected data, though the response from investigators has been mixed. A few institutions such as the Allen Institute for Brain Science, the Child Mind Institute, INRIA, Janelia Research Campus, and the Montreal Neurological Institute have made open science a core component of their values. Still, it will take time for traditional academic institutions to follow suit and recognize open science contributions in their tenure, promotion, and degree-granting processes. Journals are helping through the creation of publication formats that explicitly recognize data generators for sharing (e.g., Data Descriptor and Resource formats), though their consideration as a measure of academic impact for decisions regarding academic promotion has yet to be documented.

In the long term, creating an incentive structure that sustains open science will likely require a profound overhaul of the accreditation and financial models that currently control career advancement, scientific publishing, and the review process. To even engage this prospect, it is important to evaluate the primary argument in defense of open science, that science benefits from open sharing and reuse. A recent bibliometric analysis published in *Nature Communications* by the International Neuroimaging Data-sharing Initiative (INDI) team provided direct evidence of the positive impact of open sharing on brain imaging research [6]. The work focused on publications using data generated by: 1) open data-sharing consortia, where contributors provided their own independently collected data for sharing (e.g., 1000 Functional Connectomes Project, Autism Brain Imaging Data Exchange), and 2) larger projects that were explicitly designed and funded for open sharing (i.e., the Nathan Kline Institute-Rockland Sample, the NIH Human Connectome Project). Openly shared data were found to both increase the scale of scientific studies conducted by data contributors and engage scientists from a broader range of disciplines than studies with closed data. The work dispelled myths that scientific findings using shared data cannot be published in high-impact journals and showed a clear benefit for young investigators (i.e., usage in masters and doctoral theses).

These demonstrations of the impact of openly shared data are particularly relevant, as they serve to validate and further motivate efforts focused on the generation of openly shared data. A growing number of large-scale open data resources are emerging in the neurosciences and psychiatry (e.g., Child Mind Institute’s Healthy Brain Network, NIH ABCD Study, Alzheimer’s Disease Neuroimaging Initiative III, NIH Human Connectome Lifespan Studies, UK Biobank). There are also a growing number of smaller datasets shared by individual investigators through INDI and other data-sharing initiatives, such as OpenfMRI/OpenNeuro; these are allowing users to address a broader range of questions in clinical and cognitive neuroscience. Open data and resources will likely expand as funding sources require investigators to agree to share as part of the grant. The European Commission and 11 European research funding organizations recently created such an initiative to ensure that by 2020 all articles they financially support are immediately open access upon publication, and that the same will be true of research data for participants in the Horizon 2020 Open Research Data Pilot.

The reality is that the vast majority of scientists participate in some form of open science every day—it is just a matter of whether we are a contributor, a user, or both, and how actively. While the open science ideal would be that we would all fully operate as contributors and users, it is important to acknowledge that participation in any category makes a difference. Contributors create opportunities for others to inspect, evaluate, and build upon their contributions, yielding new outputs that may not have ever been imagined. Users help to improve the quality, reproducibility, and standardization of science simply by using common resources.

Looking forward, it makes sense to focus on increasing the benefits individuals receive through their participation in open science. A continued emphasis on growing the breadth, sophistication and usability of open tools will help to increase their use. Similarly increasing the range, scale, quality and ease of access of open datasets will increase their representation in research. The greater challenges are clearly that of increasing motivations for contributing to open science. Each of the key stakeholders (e.g., funding agencies, institutions, journals) must meaningfully increase incentives and rewards for open science; and when insufficient, consider the implementation of enforceable mandates. Funding agencies can do their part by increasing the number of grants dedicated to the development and expansion of infrastructural support for open science; the BRAIN initiative funding opportunities focused on data archived, analysis software, and data standards are excellent examples, though more are needed. Equally important is the need for more funding opportunities explicitly focused on the creation of open data resources for immediate sharing; these would value the potential significance, innovation, and quality of proposed data, rather than the specific analyses the data contributor chooses to perform. They could also motivate sharing by down-prioritizing proposals by investigators with a history of non-compliance with sharing requirements. Academic institutions have an opportunity to push the balance in favor of sharing by revising the degree-granting, promotion, and tenure processes to explicitly encourage and reward successful sharing. Journals can more uniformly implement and enforce requirements for sharing.

An obvious question that may arise is whether the current leaders and power holders in science will make significant changes in support of open science, given the inertia that has dominated to date. Such skepticism is understandable, but it is important to note the generational shift underway. Younger researchers, whose work has most directly benefited from open science, are slowly becoming the newest generation of leaders and reviewers. These individuals appear more willing to embrace the principles of open science than their predecessors and will have the opportunity to bring about reform. Although slow, such generational shifts have many precedents for overhauling flawed systems throughout history.

Finally, it is worth addressing the question of what we each can do, individually. We would suggest that at a minimum each of us can: (1) look for and seize opportunities, big or small, to increase our role in the open ecosystem, and (2) increase our emphasis on maximizing the quality and reproducibility of our outputs through the adoption of common data collection, storage, and analysis standards, even if we do not intend to share at the present time. To help make these calls to action more concrete, we include a starter list in Table 1 for actionable steps that individuals can take in pursuit of these goals. We will continue to update these steps with the help of the community at https://matter.childmind.org/open-science.html.

**Table 1:** Ways that researchers can promote the practice of open science today

• Within one’s home institution ° Catalyze open science practices through seminars, workshops, hackathons, contests [7]. ° Join groups that advocate evaluation or promotion criteria in support of open science. ° Pursue funding opportunities that require or permit open intellectual property. ° Opt for open methods rather than proprietary, licensed products. ° Strive toward reproducibility. ° Apply liberal licenses to documents and software. ° Store data in free and open access repositories. • Collaborations ° Forge ties across labs to share resources. ° Collaborate with institutions that require open standards. ° Use collaborative software and collaborative software engineering practices. ° Publish a code of conduct for each project to clarify roles and to help resolve disputes. ° Clarify contributor roles at the outset of a project to assign appropriate credit and accountability, especially for open contributions. ° Clarify when contributions to a project can be released. ° Avail oneself of experts in alternative and complementary methods to reduce bias [8], evaluate methods, and corroborate results. ° Participate in interdisciplinary open science and collaboration events. • Publications and presentations ° Preregister research, and openly publish the preregistration. ° Encourage participation of scientists and non-scientists alike. ° Publish and present in venues and in accessible language intended for general audiences, and audiences of different disciplines. ° Publish in open access venues and follow FAIR (findable, accessible, interoperable, reusable) principles. ° Publish in open data and open methods journals. ° Follow community-supported data format and reporting guidelines. ° Insist on publishing experimental protocols and negative results. ° Boycott review or submission for publishers and publications that flout open standards. ° When reviewing others’ work, acknowledge attempts and provide recommendations toward more open science practices. ° Participate in open peer review, especially in languages other than English. ° Include an ethics section to articulate ethical considerations and implications. ° Study and report the costs and benefits of your own open practices. ° Make it clear where people can access open resources that you mention. ° When someone else mentions a resource, ask about access and usage restrictions. ° Include open resources on one’s webpage and CV. • Social media ° Use and contribute to wikis and social Q&A networks. ° Do not engage in ad hominem attacks. ° Do not take others’ comments personally; respond to the science and request guidance toward better open science practices. ° Tactfully ask clarifying questions to help guide a discussion toward a useful resolution. ° Publicly acknowledge contributions to open science projects whenever possible.	

“We but mirror the world,” Gandhi said. “If we could change ourselves, the tendencies in the world would also change.” If we each faithfully do our part, the collective will effect change in science from the bottom up.
